# A Case of De Novo Antiglomerular Basement Membrane Disease Presenting during Pregnancy

**DOI:** 10.1155/2021/5539205

**Published:** 2021-03-17

**Authors:** F. A. K. Lodhi, T. Akcan, J. N. Mojarrab, S. Sajjad, R. Blonsky

**Affiliations:** ^1^Department of Internal Medicine, Marshfield Clinic Health System, Marshfield, WI, USA; ^2^Department of Pathology, Marshfield Clinic Health System, Marshfield, WI, USA; ^3^Department of Nephrology, Marshfield Clinic Health System, Marshfield, WI, USA

## Abstract

*Background*. Acute kidney injury (AKI) requiring dialysis during pregnancy is uncommon. We present a case of a young female diagnosed with antiglomerular basement membrane (anti-GBM) disease during pregnancy. *Case Presentation*. A 23-year-old woman approximately 15 weeks pregnant experienced weakness, nausea, vomiting, and anorexia for one week and anuria for 48 hours. No known drug allergies and no significant social or family history for kidney or genitourinary disease were reported. Laboratory analysis revealed anemia, life-threatening hyperkalemia, AKI, and elevated antiglomerular basement membrane (GBM) antibodies. Renal biopsy revealed 100% cellular crescents, confirming the diagnosis. The patient was treated using plasmapheresis and methylprednisolone followed by oral steroids, azathioprine, and tacrolimus. At 24 weeks and 4 days of gestation, the patient had hypoxic respiratory failure as well as preterm premature rupture of membranes. Due to the development of infection and lack of renal recovery, immunosuppression was discontinued. At 28 weeks and 0 days of gestation, the patient developed uncontrollable hypertension requiring emergent delivery. Postpartum, her hypertension improved without signs of preeclampsia though still requires dialysis. *Discussion*. Pregnancy presents a unique challenge for providers treating patients with anti-GBM disease. Fetal safety should be considered and risks thoroughly discussed with the patient when choosing an immunosuppressive regimen for this condition.

## 1. Introduction

Antiglomerular basement membrane (anti-GBM) disease is a rare, life-threatening, small vessel vasculitis usually presenting as rapidly progressive glomerulonephritis characterized by glomerular cellular crescents and linear deposits of IgG along the GBM [1]. Anti-GBM disease is reported to have an incidence of less than 1 per million population per year [2]. Standard therapy today consists of a combination of plasmapheresis, corticosteroids, and immunosuppressive drugs [3]. Levy et al. [4] reported that patients with anti-GBM disease who are dialysis-dependent on presentation showed only 8% renal survival at 1 year despite immunosuppression and plasma exchange. Given its rarity, there are only a handful of case reports documenting the occurrence of anti-GBM disease during pregnancy, treatments offered and long-term follow-up revealing the outcome of pregnancy, or the disease process itself, as a majority of pregnancies are terminated prior to term delivery to prevent worsening of conditions [5–8].

Here, we present a young woman diagnosed with anti-GBM disease early in her pregnancy and later giving birth prematurely with pre- and postpartum maternal, fetal, and neonatal course of events.

## 2. Case Presentation

A 23-year-old previously healthy woman at 15 weeks and 3 days gestation of her third pregnancy presented to the emergency department with complaints of persistent watery diarrhea, nausea, and vomiting for one week, bilateral lower extremity numbness, tingling, and weakness for five days, and anuria for 48 hours. Her past medical history was significant for obesity with a body mass index of 37.2 kg/m^2^, vaginal delivery of a healthy female infant at 41 weeks of gestation 10 months prior to the current presentation, and a first-trimester miscarriage six months prior to presentation. Her medications included prenatal vitamins, folic acid, vitamin C, and ondansetron for nausea. She denied alcohol intake, tobacco use, or substance abuse. She had no known drug allergies. There was no previous history of renal or pulmonary disease. No family disease was known and she had no other relevant findings in her medical history.

The patient was afebrile on presentation with a blood pressure of 114/53 mmHg, a heart rate of 68 beats/minute, a respiratory rate of 31 breaths/minute, and an oxygen saturation of 98% on room air. The remainder of her systemic examination findings, including those for her chest, were within normal limits.

Initial laboratory workup included a complete blood count and comprehensive metabolic panel which showed anemia and electrolyte imbalance. Blood cell analysis revealed a normal total protein level, white blood cell count of 10.1 × 10^3^ *μ*L, mean corpuscular volume (MCV) of 72 fL, low serum albumin of 3.1 g/dL, hemoglobin level of 7.8 g/dL, and hematocrit of 24.1 % and elevated red cell distribution width (RDW). Electrolyte and metabolite analysis revealed low sodium level (129 mmol/L) and elevated potassium, creatinine, and blood urea nitrogen (BUN) levels of 7.1 mmol/L, 19.8 mg/dL, and 113 mg/dL, respectively, with a decreased estimated glomerular filtration rate (eGFR) of 2.2 mL/min compared to normal values from a metabolic profile checked six months prior and an elevated hemoglobin level of 12.3 g/dL one month prior to presentation. Transaminases were within normal limits. Urinalysis showed hematuria with >100 red blood cells per field and proteinuria with 100 mg/dL protein. Furthermore, the patient was found to have an elevated erythrocyte sedimentation rate (ESR) of 117 mm/hr and mildly elevated haptoglobin of 298 mg/dL. Hematological analysis and workup for evidence of disseminated intravascular coagulation revealed a normal (INR) of 1.0, activated partial thromboplastin time (APTT) of 36.0 seconds, and normal platelet response yet elevated levels of fibrinogen (798 mg/dL) and D-dimer (2,461 ng/mL). Iron panel detected a normal serum iron level of 46 *μ*g/dL and low total iron-binding capacity (TIBC) of 225 *μ*g/dL with a corresponding transferrin saturation percentage of 20%, reticulocyte count of 25.5 × 10^2^ *μ*L, and reticulocyte percentage of 0.78.

Further laboratory workup for autoimmune conditions, infectious disease, or history of drug use revealed elevated levels of serum anti-GBM antibodies at 7.6 units (U)/mL and no evidence for underlying microbial infection or drug-induced toxicity. Serum samples were negative for cryoglobulins, antiphospholipid, antinuclear, and cytoplasmic antibodies as well as extractable nuclear antigen and antibodies to red blood cells or ADAMS TS 13. Complement levels (C3 and C4) were within normal limits.

A renal ultrasound was performed and measured the right kidney at 14.6 × 7.7 × 7.8 cm and 13.7 × 7.2 × 7.2 cm for the left kidney and increased resistance pattern in the renal vasculature with no evidence of hydronephrosis, renal calculi, or focal lesions. The bladder appeared normal. No cause of acute renal failure was identified by diagnostic imaging. Obstetrical extended ultrasound revealed a single, living intrauterine gestation at 15 weeks and 3 days based on menstrual dates and fetal biometric measurements. Fetal growth was found to be appropriate for gestational age. Anatomic survey was limited due to early gestational age but overall appeared to be unremarkable. The fetus was found to be active in normal amniotic fluid volume. No CT chest was obtained on the initial presentation due to the absence of pulmonary symptoms and in an effort to avoid radiation exposure.

**Figure 1:**
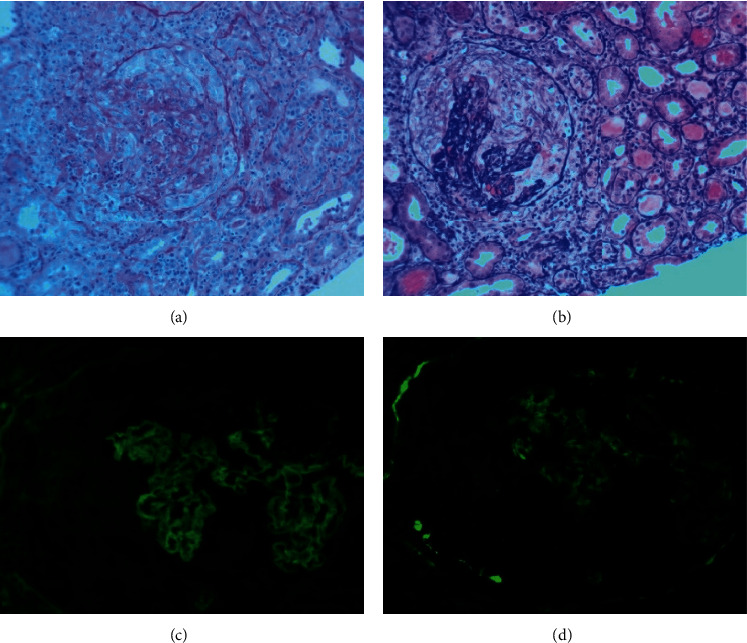
27 glomeruli were seen on the renal biopsy specimen with the following histological findings. (a) Fibrocellular crescents in the glomeruli and lymphocyte infiltration around the tubule-interstitium near the glomerulus (periodic acid-Schiff (PAS) staining ×200). (b) Glomerulus with large crescent and collapsed tuft (Jones Silver staining ×200). (c, d) Immunofluorescence staining revealed linear staining along the glomerular capillary loops (IgG staining ×400).

The patient received hemodialysis on day one via the right internal jugular temporary hemodialysis catheter placed emergently in ICU. The urgent need for pathological diagnosis and its associated risk to the mother and the fetus was thoroughly discussed with the patient and obstetrics/gynecology with the decision made to proceed with renal biopsy with continuous fetal monitoring and administration of desmopressin for bleeding risk associated with acute kidney injury and uremic platelets. Renal biopsy revealed findings consistent with rapidly progressing glomerulonephritis with 100% cellular crescents and immunofluorescence exhibiting diffuse linear staining along the glomerular basement membrane with IgG (3+), C3 (3+), kappa (3+), and lambda (2+) stains (Figures 1(a)–1(d)). Electron microscopy supported light microscopy findings of a diagnosis of anti-GBM disease. The patient underwent intermittent hemodialysis six days a week, four hours each (24 hours/week) to maintain electrolytes and laboratory numbers as close to normal as possible with pregnancy. Plasmapheresis was done for a total of seven treatments via the same right internal jugular access as used for hemodialysis. 1 plasma volume was exchanged, and for replacement, 5% albumin was used. However, for initial treatment, two units of fresh frozen plasma were used as it was the day following renal biopsy. Blood flow was 85 mL/min with a schedule of two days on with one day off to allow for clotting factors to reform. No anticoagulation was used for in-patient hemodialysis, and citrate with calcium infusion was used during plasmapheresis. She was treated with 1 g intravenous methylprednisolone every three days followed by oral prednisone daily with a slow taper, azathioprine 50 mg oral daily with tapering to a goal dose of 150 mg daily after a thiopurine methyltransferase (TPMT) enzyme assay tested negative, and 3 mg tacrolimus every 12 hours with a goal trough level of 3–7. Pneumocystis carinii pneumonia prophylaxis was also initiated with atovaquone. Other treatments included intravenous ferric gluconate for a total of five doses followed by darbepoetin alfa weekly. Renal function was monitored during the patient's hospital stay which showed mild improvement in creatinine, but unfortunately, she continued to be dialysis-dependent. A tunneled dialysis catheter was placed, and the patient was discharged in a stable condition after a 10-day hospital stay with the fetus at a gestational age of 16 weeks and 6 days. The patient followed up with her primary care physician, nephrologist, and obstetrician in the outpatient setting and remained stable on hemodialysis and immunosuppressive regimen until two months later when she was admitted again with preterm premature rupture of membranes at 24 weeks and 4 days of gestation. The patient had experienced shortness of breath and chest tightness for several weeks which were considered to be secondary to her pregnancy.

**Figure 2:**
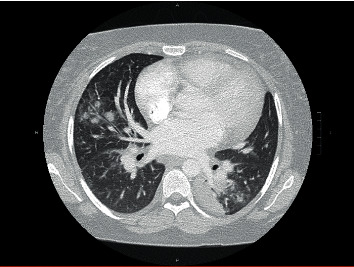
Computed tomography pulmonary angiogram showing no pulmonary embolism; multifocal pulmonary infiltrates; small right-sided pleural effusion; very small pericardial effusion; cardiomegaly.

Given concerns for shortness of breath and chest tightness, a chest X-ray and chest computed tomography (CT) scan (Figure 2) were performed and were negative for pulmonary embolism though she developed bilateral patchy infiltrates of undetermined etiology while being on chronic immunosuppression for the last two months with tacrolimus, azathioprine, and prednisone. She was transferred to the critical care unit for airway monitoring. On admission, her systolic blood pressure was elevated at 130 mmHg, and her oxygen saturation rose to 96% on 6 L via oxygen mask. Medication history included tacrolimus, azathioprine, nifedipine, labetalol, prednisone, progesterone vaginal suppositories, and six times per week hemodialysis. Physical examination revealed 2+ pedal edema bilaterally, and fetal examination revealed baseline fetal heart tones of 150 s, moderate variability, no decelerations, and cephalic presentation. The serum microbial panel for hepatitis B surface antigen, rubella, syphilis, gonococcal, chlamydia, and HIV was negative. The respiratory viral panel was positive for parainfluenza virus. The patient was continued on antibiotics with symptomatic management for parainfluenza infection.

Later, the patient developed severe hypertension which was not manageable with antihypertensive drugs, and the decision was made to deliver the fetus. Emergent cesarean section was performed at 28–0/7 weeks, and a male neonate, 1.1 kg with Apgar scores of 1/6/7, was delivered. The patient's postpartum course was relatively uncomplicated with no development of postpartum preeclampsia. The patient remained on hemodialysis until the end of her postpartum period, following which she transitioned to peritoneal dialysis and is currently awaiting a transplant.

## 3. Discussion

Anti-GBM antibody disease during pregnancy is a very rare occurrence. There are a few case reports in the literature with biopsy-proven anti-GBM disease and varied presentations. The outcomes of infants from mothers with anti-GBM disease during pregnancy are generally poor, often resulting in stillbirth and both natural and artificial abortion [5–8]. Moreover, to date, only five cases resulted in successful delivery which are summarized as follows.

Deubner et al. [9] described a case of a pregnant woman with rapidly progressive glomerulonephritis (RPGN) secondary to anti-GBM disease which flared postpartum with the development of precipitous renal failure. The patient experienced a full-term pregnancy and gave birth to a healthy infant. Deubner et al. postulated that the placenta may have served as an adsorptive surface for the autoantibody and may have ameliorated her symptoms. Yankowitz et al. [10] reported the case of a 28-year-old woman who was diagnosed with anti-GBM disease three months prior to her pregnancy and was treated with intensive hemodialysis and corticosteroids along with cyclophosphamide and who delivered an infant at 37 weeks of gestation. The patient's anti-GBM antibody levels became negative during pregnancy and were detected again after delivery. Al Harbi et al. [11] reported a case of a 30-year-old woman who presented with acute renal failure at 28 weeks of gestation and was treated with antibiotics, intensive hemodialysis, and corticosteroids and delivered an infant at 34 weeks of gestation. Sprenger-Maehr et al. [12] also described a case of a 30-year-old woman with anti-GBM disease who was treated with plasma exchange, corticosteroids, cyclophosphamide, and rituximab and delivered an infant at 38 weeks of gestation. Lastly, Vasiliou et al. [13] reported a 34-year-old woman who presented with RPGN and was diagnosed with anti-GBM disease during 18 weeks of gestation. She was treated with hemodialysis, intermittent plasmapheresis, and immunosuppressive therapy with tapering steroids and azathioprine. In all cases, reduction of anti-GBM antibodies via placental adsorption or combination immunosuppressive treatment and hemodialysis appears to improve pregnancy outcome.

The present case is unique in terms of diagnosis, flare-up, and treatment for anti-GBM disease during the course of pregnancy followed by a successful preterm delivery. In our case, the patient presented with anti-GBM disease during the early phase of her third pregnancy with no evidence of kidney dysfunction during her previous pregnancies. The anti-GBM antibody titers were very high at the time of diagnosis and unlike the cases reported by Deubner et al. [9] and Yankowitz et al. [10] did not become negative after treatment during pregnancy or 5 months postpartum. Our patient experienced a precipitous decline in renal function with only moderate improvement with immunosuppression along with intensive plasma exchange and hemodialysis. Treatment with immunosuppressive agents such as tacrolimus and azathioprine in addition to steroids allowed us to defer administration of cyclophosphamide use and resulted in a successful delivery. To our knowledge, this is the first report in the literature of a woman diagnosed with anti-GBM disease during early pregnancy who was treated with a combination of immunosuppressive drugs tacrolimus and azathioprine to result in a successful delivery.

Maternal, pregnancy, and fetal outcomes of anti-GBM disease in pregnancy are poor and are associated with uncontrolled vasculitis [5–8]. The treatment of choice in anti-GBM antibody disease is intensive plasmapheresis combined with immunosuppressive agents such as steroids, cyclophosphamide, and azathioprine [3, 4]. Plasmapheresis removes circulating anti-GBM antibodies and other mediators of inflammation while immunosuppressive agents minimize new antibody formation. Plasmapheresis is generally considered safe in pregnancy [14]. Systemic corticosteroids are generally considered low risk for teratogenicity although there are historical associations of this regimen to cleft palate [15]. Cyclophosphamide is the main immunosuppressive drug that has been used in anti-GBM disease for over 30 years. The challenge arises in pregnant patients where the literature does not support the use of cyclophosphamide, as there is a risk of teratogenicity, especially in the first trimester [16]. Growth retardation, suppression of fetal hematopoiesis, and impaired neurological development have been reported with use later in pregnancy in the second and third trimesters [17, 18]. Cyclophosphamide is excreted in breast milk and is also contraindicated during breastfeeding [19]. The use of rituximab for the treatment of anti-GBM disease has also been explored in the medical literature. However, limited data exist, but the use of this B-cell depleting agent may be considered as a viable option in anti-GBM disease that is refractory to standard treatment [20]. Pregnancy complicates its use, and after a detailed discussion regarding risks and benefits, the decision was made to not proceed with this agent due to maternal concerns of fetal B-cell depletion and other hematological abnormalities [21]. The use of tacrolimus and azathioprine is compatible in pregnancy and breastfeeding as per current literature [16, 22, 23]. Our patient presented in the second trimester during which time the use of cyclophosphamide is acceptable, but due to the lack of sufficient evidence regarding renal recovery with its use in anti-GBM disease, the decision was made to proceed with tacrolimus and azathioprine. The present case is an example that successful deliveries can be achieved in pregnant patients presenting with anti-GBM who are treated with immunosuppressive agents such as tacrolimus and azathioprine in addition to steroids; moreover, use of these agents may permit deferral of cyclophosphamide use until after pregnancy.

## 4. Conclusion

Anti-GBM disease should be considered in pregnant patients presenting with oligoanuric renal failure. Plasmapheresis and immunosuppression remain the treatments of choice. When choosing an immunosuppressive regimen in pregnancy, fetal safety should be considered and risks thoroughly discussed prior to treatment.

## Data Availability

The data used to support the findings of this study are from the electronic medical record, which is unavailable for public viewing.
